# The Beneficial Effect of Melatonin in Brain Endothelial Cells against Oxygen-Glucose Deprivation Followed by Reperfusion-Induced Injury

**DOI:** 10.1155/2014/639531

**Published:** 2014-07-14

**Authors:** Juhyun Song, So Mang Kang, Won Taek Lee, Kyung Ah Park, Kyoung Min Lee, Jong Eun Lee

**Affiliations:** ^1^Department of Anatomy, Yonsei University College of Medicine, Seoul, Republic of Korea; ^2^BK21 Plus Project for Medical Sciences and Brain Research Institute, Yonsei University College of Medicine, 50 Yonsei-ro, Seodaemun-gu, Seoul 120-752, Republic of Korea; ^3^Department of Neurology, Seoul National University College of Medicine, Seoul, Republic of Korea

## Abstract

Melatonin has a cellular protective effect in cerebrovascular and neurodegenerative diseases. Protection of brain endothelial cells against hypoxia and oxidative stress is important for treatment of central nervous system (CNS) diseases, since brain endothelial cells constitute the blood brain barrier (BBB). In the present study, we investigated the protective effect of melatonin against oxygen-glucose deprivation, followed by reperfusion- (OGD/R-) induced injury, in bEnd.3 cells. The effect of melatonin was examined by western blot analysis, cell viability assays, measurement of intracellular reactive oxygen species (ROS), and immunocytochemistry (ICC). Our results showed that treatment with melatonin prevents cell death and degradation of tight junction protein in the setting of OGD/R-induced injury. In response to OGD/R injury of bEnd.3 cells, melatonin activates Akt, which promotes cell survival, and attenuates phosphorylation of JNK, which triggers apoptosis. Thus, melatonin protects bEnd.3 cells against OGD/R-induced injury.

## 1. Introduction

Stroke is the third most frequent worldwide cause of adult death [1, 2]. Specifically, about 80% of all strokes are ischemic, resulting from arterial occlusion in the brain [1]. Reperfusion after occlusion results in serious brain injury, due to overproduction of reactive oxygen species (ROS), calcium overload [3, 4], and blood-brain barrier (BBB) injury [5]. Finally, in ischemic stroke, the brain is damaged because of hypoxia and oxidative stress [6–10]. Reactive oxygen species (ROS) play a key role in the pathogenesis of many diseases, including central nervous system (CNS) diseases [11–14]. During ischemic stroke, the excessive generation of ROS leads to inflammation and cell apoptosis [15–21] and induces mitogen-activated protein kinase (MAPK) signaling [22–24]. c-Jun N-terminal kinase (JNK), one of the MAPKs, is activated by a variety of cell stresses, including hyperosmotic shock, hypoxia, and ROS [25, 26]. JNK plays key roles in apoptosis and inflammation [27, 28]. JNK signaling is activated by inflammatory cytokines and promotes neuronal cell death [29]. Endothelial cells are also damaged by activation of JNK signaling, in response to oxidative stress [30]. Several studies have demonstrated that, in hypoxia and a state of reoxygenation, cells induce apoptotic signaling through JNK and p38 MAPK [31, 32]. The BBB controls the exchange of materials between blood and the brain and plays an important role in the homeostatic regulation of the brain microenvironment [33]. The tight junctions between capillary endothelial cells, which form an essential structural component of the BBB [34], include membrane proteins like occludin [35] and claudins [36, 37]. Several studies have suggested that hypoxia causes alterations of the tight junction proteins Claudin 5, occludin, ZO-1, and ZO-2, which affect BBB permeability [38, 39]. In addition, vascular endothelial growth factor (VEGF) is an inducer of vascular leakage [40] and is also known as vascular permeability enhancing factor [41, 42]. During ischemia, VEGF interacts with receptors for VEGF on the ischemic vessels and contributes to disruption of the BBB [43, 44]. Zhang el al. demonstrated that inhibition of VEGF reduces BBB permeability [43]. Melatonin is synthesized in the pineal gland and has been known to function as an antioxidant [45]. Melatonin reduces the cellular toxicity of ROS in ischemia and reperfusion (I/R) brain injury [46]. In an* in vivo* cerebral ischemia model, several researches have demonstrated that melatonin treatment reduces brain damage in the setting of ischemia or hypoxia-induced injury [47, 48].* In vitro, *melatonin protects primary neuronal cells from apoptotic death [49] and enhances survival of human neuroblastoma cells [50] in the setting of oxygen-glucose deprivation- (OGD-) induced injury. Furthermore, melatonin suppresses VEGF expression in cancer cells [51, 52] and inhibits serum VEGF levels in patients [53]. In the present study, we investigate whether melatonin protects brain endothelial cells against oxygen-glucose deprivation followed by reperfusion- (OGD/R-) induced injury. We show that melatonin reduces the generation of ROS, prevents disruption of the BBB by stabilizing expression of tight junction proteins and suppressing VEGF expression, and attenuates phosphorylation of JNK, a mediator of cellular apoptosis. Therefore, our results suggest that melatonin is important in protecting the BBB against cerebral ischemic damage.

## 2. Materials and Methods

### 2.1. Cell Culture

Murine brain endothelial cells (bEnd.3 cells; ATCC, VA, USA) were purchased from ATCC and cultured in Dulbecco's modified Eagle's medium (DMEM, Hyclone Laboratories, UT, USA), supplemented with 10% (v/v) fetal bovine serum (FBS, Hyclone Laboratories, UT, USA) and 100 units/mL of penicillin/streptomycin (Hyclone Laboratories, UT, USA), at 37°C in a humidified atmosphere in the presence of 5% CO_2_ [54]. bEND.3 cells were used at 13 passages in this study.

### 2.2. Oxygen-Glucose Deprivation (OGD) and Reperfusion

Confluent cells were transferred to an anaerobic chamber (Forma Scientific, OH, USA) (O_2_ tension, 0.1%) and washed three times with PBS. Then, culture medium was replaced with deoxygenated, glucose-free balanced salt solution, and cells were incubated for 6 h. Following oxygen-glucose deprivation (OGD) injury, cells were incubated for 18 h under normal growth conditions, with or without drug treatment [55].

### 2.3. Drug Treatment

Melatonin was purchased from Sigma (Sigma, MO, USA) and dissolved in ethanol. An equivalent volume of ethanol (final: 0.01%) or water was added to control and all melatonin-containing wells. bEnd.3 cells were exposed to 1–100 nM melatonin for 24 h before OGD/R injury. The present study consisted of four groups: (1) normal control (NC), bEnd.3 cells cultured with normal media without OGD injury; (2) experimental control (EC), bEnd.3 cells cultured in nontreated medium for 18 h after 6 h of OGD injury; (3) 10 nM melatonin (Mel 10 nM), bEnd.3 cells treated with 10 nM melatonin for 24 h before 6 h of OGD injury; these cells were then cultured in nontreated medium for 18 h; (4) 100 nM melatonin (Mel 100 nM): bEnd.3 cells were also treated with 100 nM melatonin (100 nM melatonin group) for 24 h before 6 h of OGD injury. These cells were then cultured in nontreated medium for 18 h. In Akt inhibitor groups, we treated 100 nM Akt inhibitor (Sigma, MO, USA) together with melatonin.

### 2.4. Hoechst 33258 and Propidium Iodide (PI) Staining

Cell viability was evaluated by staining bEnd.3 cells with Hoechst 33258 dye (Sigma, MO, USA) and propidium iodide (PI; Sigma, MO, USA). Hoechst dye was added to the culture medium (2-3 *μ*g/mL) and samples were then incubated at 37.8°C for 30 min. PI solution was then added (2–5 *μ*g/mL) just before cells were observed with a microscope (BX51; Olympus) equipped with epifluorescence and a UV filter block. PI-positive cells were counted as dead cells [56].

### 2.5. Cell Viability Assay

bEnd.3 cells (2 × 10^5^ cells/mL) were seeded in 98-well plates to monitor all experiment conditions, including pretreatment, OGD injury, and reperfusion. Next, cells were rinsed twice with phosphate-buffered saline (PBS), and culture medium was replaced with serum-free medium and 100 *μ*L 3-[4,5-dimethylthiazol-2-yl]-2,5-diphenyl tetrasodium bromide (MTT) (Sigma, MO, USA) solution (5 mg/mL in PBS) per well. After 1 h of incubation, medium was removed and dimethyl sulfoxide (DMSO) was added to solubilize the purple formazan product of MTT treatment. The supernatant from each well was analyzed using an ELISA plate reader (Labsystems Multiskan MCC/340; Fisher Scientific, PA, USA) at a wavelength of 570 nm, with background subtraction at 650 nm. All experiments were repeated at least three times. Cell viability in the control medium, without any treatment, was represented as 100%. Cell viability was reported as a relative value, compared to the control group.

### 2.6. Lactate Dehydrogenase (LDH) Assay

Cytotoxicity in all treatment groups was quantified by measuring the amount of LDH released into the culture medium from OGD/R-injured cells [57, 58]. LDH release (cytotoxicity %) was calculated by dividing the value at the experimental time point by the maximum value. The maximum LDH release was measured after freezing each culture at −70°C overnight, followed by rapid thawing, which induced nearly complete cell damage.

### 2.7. Determination of Intracellular ROS

The level of intracellular ROS in each treatment group was measured using a fluorescent probe, 2′,7′*-*dichlorodihydrofluorescein diacetate (DCF*-*DA; Invitrogen, CA, USA), as previously described [59]. Cells were plated at a density of 1 × 10^6^ cells/mL and treated with melatonin for 24 h. After melatonin pretreatment, OGD injury and reperfusion were conducted. Then, bEND.3 cells were treated with 5 *μ*M DCF-DA for 30 min at 37°C. After washing with PBS, fluorescence was measured with a microscope (Nikon TS100-F ECLIPSE) equipped with a CCD camera (Hamamatsu Photonics) [54].

### 2.8. Western Blot Analysis

After pretreatment, OGD injury, and restoration, cells were washed rapidly with ice-cold PBS, scraped, and collected. Cell pellets were lysed with ice-cold RIPA buffer (Sigma, MO, USA). The lysates were centrifuged at 13,200 rpm for 1 h at 4°C to produce whole-cell extracts. Protein content was quantified using the BCA method (Pierce, IL, USA). Protein (20 *μ*g) was separated on a 10% SDS-polyacrylamide (PAGE) gel and transferred onto a polyvinylidene difluoride (PVDF) membrane. After blocking with 5% bovine serum albumin, prepared in Tris-buffered saline/Tween (TBS-T; 20 nM Tris (pH 7.2); 150 mM NaCl; 0.1% Tween 20), for 1 h at RT, immunoblots were incubated overnight at 4°C with primary antibodies that specifically detect Akt (1 : 2000, Cell Signaling, MA, USA), p-Akt (1 : 2000, Cell Signaling, MA, USA), JNK (1 : 2000, Cell Signaling, MA, USA), p-JNK (1 : 2000, Cell Signaling, MA, USA), Claudin 5 (1 : 1000, Santa Cruz, CA, USA), VEGF (1 : 1000, Millipore, MA, USA), Bax (1 : 2000, Cell Signaling, MA, USA), or *β*-actin (1 : 2000, Cell Signaling, MA, USA). Next, blots were incubated with HRP-linked anti-mouse and -rabbit IgG antibodies purchased from Abcam (Cambridge, MA, USA) for 1 h at RT. Enhanced chemiluminescence was performed by ECL (Pierce, IL, USA) [54].

### 2.9. Immunocytochemistry (ICC)

The expression of VEGF and Claudin 5 in bEnd.3 cells was confirmed by immunocytochemistry. Cells in all experimental groups were washed three times with PBS, fixed with 4% paraformaldehyde for 3 h, and then washed with PBS. bEnd.3 cells were permeabilized with 0.025% Triton X-100 and blocked for 1 h at RT with dilution buffer (Invitrogen, CA, USA). Primary anti-rabbit VEGF (1 : 500, Millipore, MA, USA) and anti-rabbit Claudin 5 (1 : 500, Santa Cruz, CA, USA) antibodies were prepared in dilution buffer, added to samples, and incubated for 3 h at RT. Primary antibody was then removed and cells were washed three times for 3 min each with PBS. Later, samples were incubated with FITC-conjugated goat, anti-rabbit (1 : 200, Jackson Immunoresearch, PA, USA) or Rhodamine-conjugated donkey, or anti-rabbit secondary antibodies (1 : 500, Millipore, MA, USA) for 2 h at RT. Cells were washed again three times for 3 min each with PBS and stained with 1 *μ*g/mL 4′,6-diamidino-2-phenylindole (DAPI) (1 : 100, Invitrogen, CA, USA) for 10 min at RT. Fixed samples were imaged using a Zeiss LSM 700 confocal microscope (Carl Zeiss, NY, USA).

### 2.10. Statistical Analysis

Statistical comparisons were performed using independent* t*-tests for two groups. SPSS software was used for all analyses. Data were expressed as mean ± S.E.M. of three independent experiments. Differences were considered significant at ^#^ *P* < 0.1, ∗*P* < 0.05, and ∗∗*P* < 0.001.

## 3. Results

### 3.1. Melatonin Attenuates the Cell Death of bEND.3 Cells after OGD/R-Induced Injury

**Figure 1:**
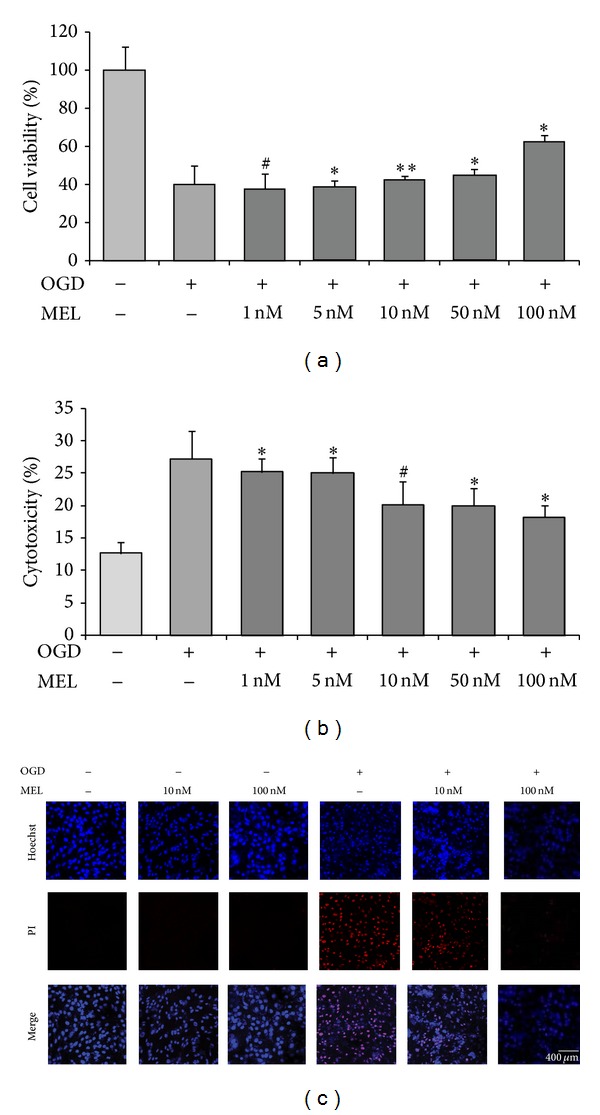
The measurement of brain endothelial cell viability after OGD/R-induced injury. (a) A 3-(4,5-dimethylthiazol-2-yl)-2,5-diphenyltetrazolium bromide (MTT) assay shows that bEND.3 cells in the OGD/R injury exposed group exhibited decreased viability compared to cells in the normal control group. Cell viability of bEND.3 cells in 1 nM and 5 nM melatonin pretreatment groups was not largely different form OGD/R injury exposed group. bEND.3 cells in 10 nM, 50 nM, and especially 100 nM melatonin pretreatment groups exhibited increased cell viability compared to OGD/R injury exposed group. Data are expressed as mean ± S.E.M. (^#^ *P* < 0.1, ∗*P* < 0.05, and ∗∗*P* < 0.001). (b) Cytotoxicity (%) was measured using an LDH assay. Cytotoxicity increased in OGD/R injury exposed group compared to the normal control group. Melatonin treatment (especially 100 nM melatonin pretreatment) reduced cytotoxicity after OGD/R injury. Data are expressed as mean ± S.E.M. (^#^ *P* < 0.1, ∗*P* < 0.05, and ∗∗*P* < 0.001). (c) Dead and live cells were measured by Hoechst/PI staining. PI-positive cells (red) are regarded as the dead cells. PI-positive cells were higher in OGD/R injury exposed group than in the normal control group. Melatonin treatment groups (both in 10 nM and in 100 nM melatonin groups) exhibited reduced PI-positive cells compared to the OGD/R injury exposed group. Hoechst: Hoechst 33342 (blue color) and PI: propidium iodide (red color). Scale bar = 400 *µ*m.

To confirm the protective effect of melatonin on OGD/R-induced injury, we first conducted an MTT assay to check cell viability in all treatment groups (Figure 1(a)). Cell viability showed that the OGD/R injury exposed group exhibited decreased cell viability, compared to the normal control group (100% cell viability in the normal control group; 39% cell viability in the OGD/R injury exposed group). We checked the cell viability by pretreatment with melatonin 1 nM to 100 nM. Cell viability in 1 nM and 5 nM melatonin pretreatment group was almost not different from the OGD/R injury exposed group. Treatment with 10 nM melatonin also did not change cell viability compared to the OGD/R injury exposed group (48% cell viability in the Mel 10 nM group). However, treatment with 100 nM melatonin obviously increased cell viability after OGD/R-induced injury, compared to the normal control group (62% cell viability in the Mel 100 nM group) (Figure 1(a)). In addition, we evaluated cytotoxicity in bEND.3 cells following OGD/R injury using an LDH assay (Figure 1(b)). Cytotoxicity was 12% in the normal control group but was 28% in the OGD/R injury exposed group. Cytotoxicity in 1 nM and 5 nM melatonin pretreatment group was not largely different from the OGD/R injury exposed group. Treating cells with 10 nM melatonin resulted in 21% cytotoxicity and treating cells with 100 nM melatonin resulted in 18% cytotoxicity (Figure 1(b)). Considering cell viability and cytotoxicity data, we decided two concentrations of melatonin (10 nM melatonin concentration (among the low concentrations: 1 nM, 5 nM, and 10 nM) and 100 nM melatonin concentration (among the high concentrations: 50 nM, 100 nM)) to compare the effect of melatonin easily. We also conducted Hoechst/PI staining to check the dead cells in all groups (Figure 1(c)). Hoechst/PI staining images showed that only melatonin treatment groups were almost not different from the normal control group. PI-positive cells (dead cells) evidently were increased in the OGD/R injury exposed group, compared to the normal control group. 10 nM and 100 nM melatonin treatment promoted cell survival and inhibited cell death against OGD/R-induced injury. In the 100 nM melatonin treatment group, the protective effect of melatonin against OGD/R injury death in bEND.3 cells was more obvious than in the 10 nM melatonin treatment group (Figure 1(c)). Taken together, these findings suggest that melatonin attenuates OGD/R-induced damage in brain endothelial cells.

### 3.2. Melatonin Decreases OGD/R-Induced ROS Production

**Figure 2:**
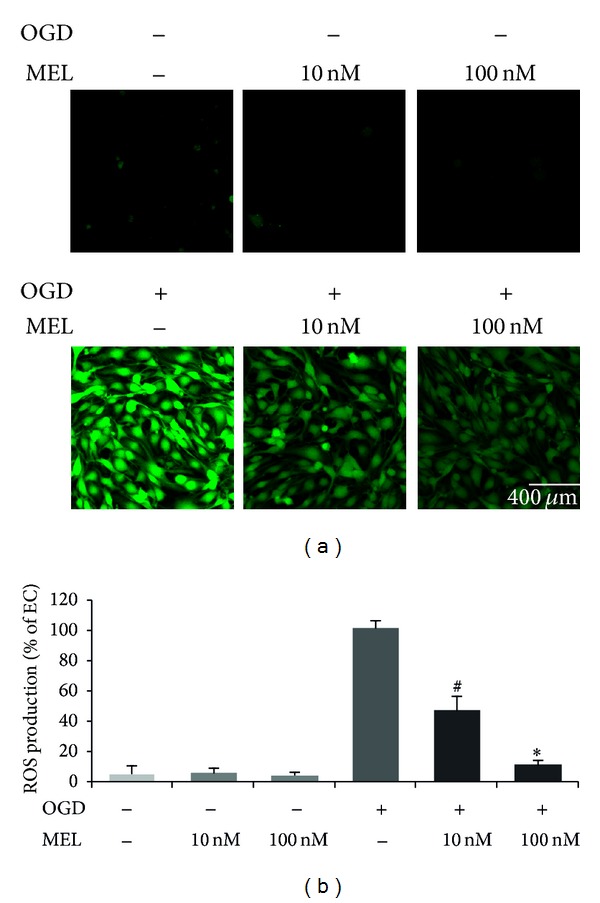
Immunocytochemistry to measure ROS generation in bEND.3 cells after OGD/R-induced injury. bEND.3 cells were treated with melatonin for 24 h before OGD/R injury. ROS levels were measured using DCF-DA. (a) ROS levels in only melatonin treatment groups (both 10 nM and 100 nM melatonin pretreatment groups) were the same as the normal control group. ROS levels in bEND.3 cells were increased in OGD/R injury exposed group. Under OGD/R injury, ROS levels in the melatonin pretreatment group were decreased compared to OGD/R injury exposed group. Melatonin decreased the OGD/R-induced increase in DCF-DA-positive cells (green). (b) ROS production was calculated by measuring the intensity of ROS. This graph shows relative intensity as a percentage of OGD/R injury exposed group. Data are expressed as mean ± S.E.M. (^#^ *P* < 0.1 and ∗*P* < 0.05). 2′,7′-Dichlorodihydrofluorescein diacetate (DCF-DA): green. Scale bar = 400 *µ*m.

We measured ROS levels using DCF-DA reagent, a fluorescent dye that visualizes ROS. DCF-DA-positive cells increased after OGD/R. ROS levels in melatonin pretreatment groups (10 nM, 100 nM melatonin) were not largely different from ROS levels in the normal control group. In the OGD/R injury exposed group, ROS levels were evidently increased compared to the normal control group. This was partially blocked by pretreatment with 10 nM melatonin (Figures 2(a) and 2(b)). 100 nM melatonin pretreatment clearly decreased the number of DCF-DA-positive cells, compared to the OGD/R injury exposed group. This result suggests that melatonin inhibits OGD/R-induced ROS production in brain endothelial cells.

### 3.3. Melatonin Prevents Degradation of Tight Junction Proteins against OGD/R Injury

**Figure 3:**
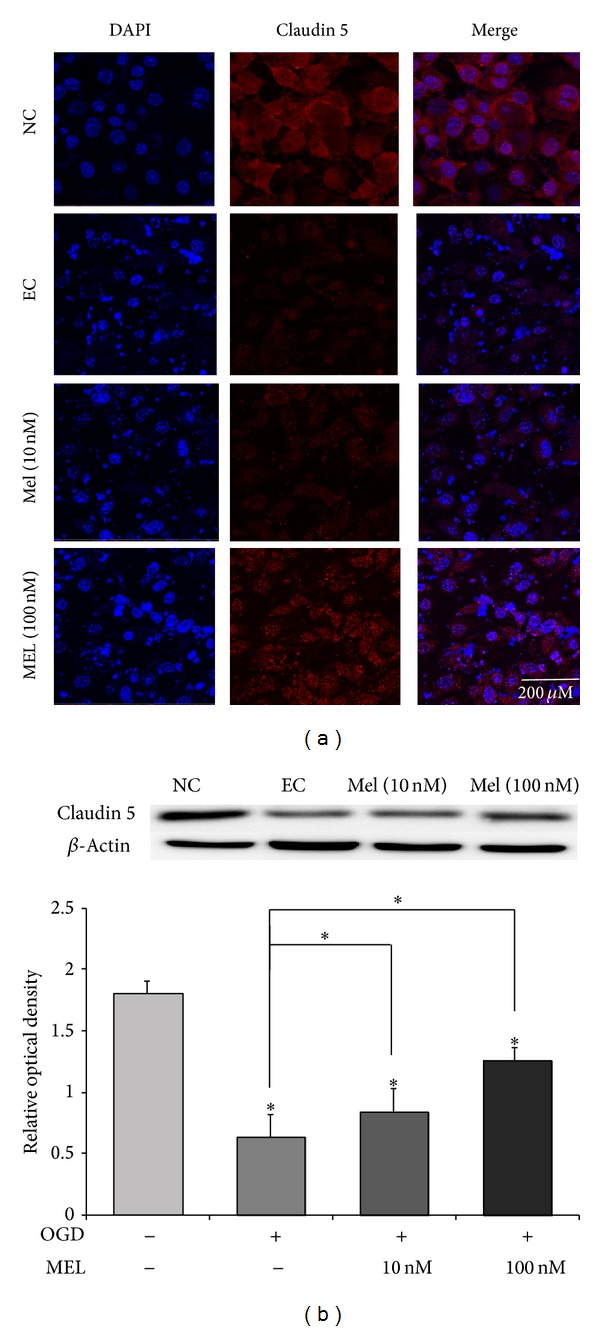
The measurement of the tight junction protein in bEND.3 cells after OGD/R-induced injury. (a) The level of Claudin 5, a tight junction protein, was evaluated by immunocytochemistry. This image shows that expression of Claudin 5 in the experimental control (EC) group decreased compared to the normal control (NC) group. Melatonin increased the expression of Claudin 5 under OGD/R injury (green). In the Mel (10 nM) and Mel (100 nM) groups, the expression of Claudin 5 was higher than in the EC group. Claudin 5 was preserved in the melatonin treatment group, following OGD/R-induced injury. Scale bar: 200 *µ*m, Claudin 5: red, and 4′,6-diamidino-2-phenylindole (DAPI): blue. (b) Western blotting showed that the relative protein level of Claudin 5 was reduced in EC compared to the NC group. The relative level of Claudin 5 was increased in Mel (10 nM) and Mel (100 nM) groups, compared to the EC group. The bar graph shows the quantification of Claudin 5 protein in all groups. *β*-Actin was used as an internal control. Data are expressed as mean ± S.E.M. (∗*P* < 0.05). (i) Normal control (NC): bEnd.3 cells cultured with normal media without OGD injury, (ii) experimental control (EC): bEnd.3 cells cultured in nontreated medium for 18 h after 6 h of OGD injury, and (iii) 10 nM melatonin (Mel 10 nM): bEnd.3 cells treated with 10 nM melatonin for 24 h before 6 h of OGD injury. These cells were then cultured in nontreated medium for 18 hr. (iv) 100 nM melatonin (Mel 100 nM): bEnd.3 cells were also treated with 100 nM melatonin (100 nM melatonin group) for 24 h before 6 h of OGD injury. These cells were then cultured in nontreated medium for 18 h.

To check the protective effect of melatonin on the integrity of tight junctions during OGD/R, we measured the level of Claudin 5, a tight junction protein, by immunocytochemistry (Figure 3(a)) and western blot analysis (Figure 3(b)). OGD/R stress obviously decreased the expression of Claudin 5 in the bEND.3 cells compared to the normal control (NC) group. The expression of Claudin 5 did not nearly change in the 10 nM melatonin treatment group, compared to the experimental control (EC) group which in exposed OGD/R injury. The expression of Claudin 5 was evidently attenuated by treatment with 100 nM melatonin (Figures 3(a) and 3(b)). This result shows that melatonin pretreatment protects degradation of Claudin 5 following OGD/R injury. Namely, melatonin may prevent deterioration of tight junctions in response to OGD/R-induced injury.

### 3.4. Melatonin Attenuates the Expression of VEGF after OGD/R-Induced Injury

**Figure 4:**
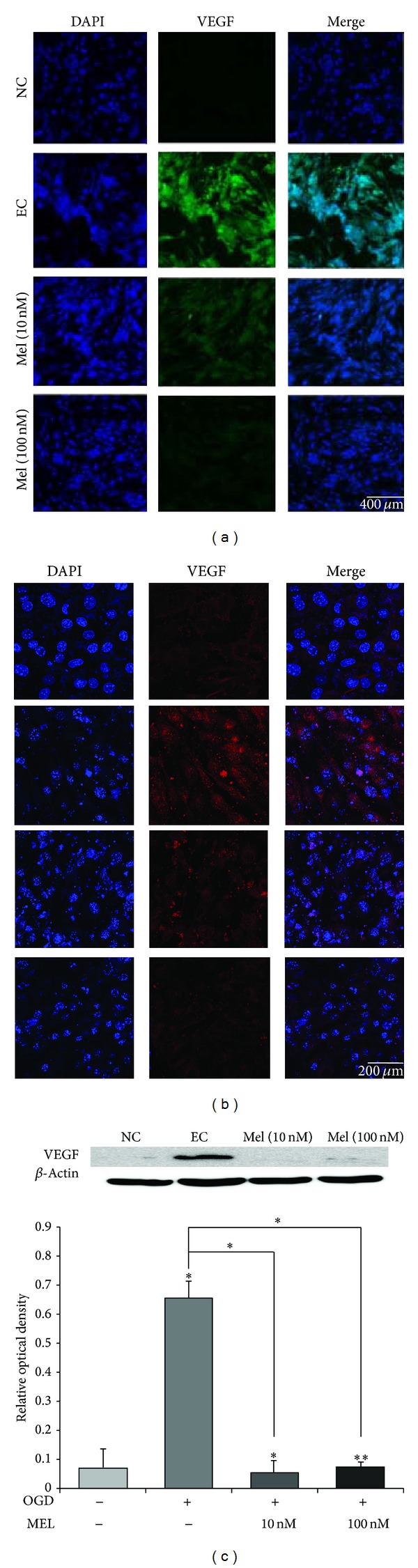
The measurement of VEGF expression in bEND.3 cells after OGD/R-induced injury. (a) The level of VEGF was evaluated by immunocytochemistry. This image shows that the expression of VEGF in the experimental control (EC) group was increased compared to the normal control (NC) group. Melatonin attenuated the OGD/R-induced increase in the number of VEGF-positive cells. In Mel (10 nM) and Mel (100 nM) groups, the expression of VEGF was lower than in the EC group. VEGF expression was attenuated in the melatonin treatment group under OGD/R-induced injury. Scale bar: 400 *µ*m. (b) Scale bar: 200 *µ*m, vascular endothelial growth factor (VEGF): green, red, and 4′,6-diamidino-2-phenylindole (DAPI): blue. (c) Western blotting showed that the protein level of VEGF was evidently increased in EC compared to the NC group. The protein level of VEGF was attenuated in both Mel (10 nM) and Mel (100 nM) groups, compared to the EC group. The bar graph shows the quantification of VEGF protein in all groups. *β*-Actin was used as an internal control. Data are expressed as mean ± S.E.M. (∗*P* < 0.05 and ∗∗*P* < 0.001).

We conducted immunocytochemistry (Figures 4(a) and 4(b)) and western blot analysis (Figure 4(c)) to confirm the expression of VEGF in all treatment groups. This result indicated that the expression of VEGF became considerably elevated after OGD/R injury in the bEND.3 cells. However, the expression of VEGF was reduced by melatonin treatment (both 10 nM and 100 nM melatonin pretreatment) (Figures 4(a) and 4(b)). This finding suggests that melatonin attenuates the expression of VEGF in brain endothelial cells following OGD/R-induced injury.

### 3.5. Melatonin Protects bEND.3 Cells via Akt Activation and JNK Suppression

**Figure 5:**
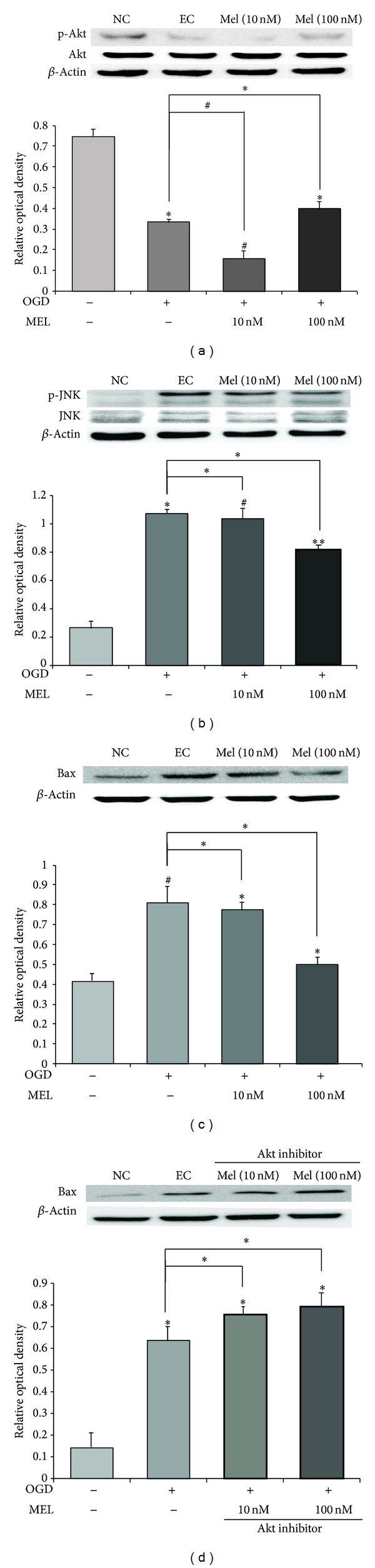
The measurement of JNK, Akt, and Bax expression in brain endothelial cells after OGD/R-induced injury. (a) Western blotting showed that the relative protein level of phosphor-Akt was reduced in EC compared to the NC group. The protein level of phosphor-Akt was increased in Mel (100 nM) groups, compared to the EC group. The bar graph shows the quantification of phosphor-Akt/Akt protein in all groups. (b) Western blotting showed that the relative protein expression of phosphor-JNK increased in the EC group, compared to the NC group. The relative level of phosphor-JNK decreased in Mel 10 nM and Mel 100 nM groups, compared to the EC group. The bar graph shows the quantification of phosphor-JNK/JNK protein in all groups. (c) Western blotting showed that the relative protein expression of Bax increased in the EC group, compared to the NC group. The protein level of Bax decreased in Mel 10 nM and Mel 100 nM groups, compared to the EC group. The bar graph shows the quantification of Bax protein in all groups. (d) Western blotting showed the relative protein expression of Bax by melatonin and 100 nM Akt inhibitor pretreatment under OGD/R injury. The expression of Bax was increased in the EC treatment group, compared to the NC group. The protein level of Bax was increased in Mel 10 nM and Mel 100 nM groups with 100 nM Akt inhibitor copretreatment, compared to the EC group. The bar graph shows the quantification of Bax in all groups. *β*-Actin was used as an internal control. Data are expressed as mean ± S.E.M. (^#^ *P* < 0.1, ∗*P* < 0.05, and ∗∗*P* < 0.001). Protein kinase B (Akt), phosphorylated Akt (p-Akt), c-Jun N-terminal kinases (JNK), and phosphorylated JNK (p-JNK).

To investigate whether Akt signaling was activated in OGD/R-induced stress, we first measured the phosphorylation status of Akt by western blot analysis (Figure 5(a)). Phosphorylation of Akt is associated with activation of Akt signaling and cell survival. Our result suggests that the protein expression of phosphor-Akt/Akt in the EC group is attenuated compared to the NC group. Expression of phosphor-Akt in the 10 nM melatonin treatment group did not nearly change compared to the EC group. However, expression of phosphor-Akt in the 100 nM melatonin treatment group was higher than in the EC group (Figure 5(a)). Next, we also examined the phosphorylation status of JNK by western blot analysis (Figure 5(b)), because the phosphorylation of JNK correlates with activation of apoptosis signaling. The expression of phosphor-JNK was decreased by melatonin treatment after OGD/R-induced injury. Pretreatment with 100 nM melatonin resulted in the obvious inhibition of JNK signaling whereas JNK activation in 10 nM melatonin pretreatment group was not largely different from the EC group (Figure 5(b)). These results suggest that melatonin 100 nM increases Akt activation and suppresses JNK activation. To confirm the relationship between melatonin and Akt signaling, we checked the expression of Bax by western blot analysis (Figure 5(c)). We confirmed that the protein expression of Bax in the bEND.3 cells was increased under OGD/R injury compared to the NC group. Also, 10 nM and 100 nM melatonin treatment reduced the protein expression of Bax under OGD/R injury. When we checked the expression of Bax in OGD/R injured bEND.3 cells with Akt inhibitor and melatonin pretreatment, we confirmed that Akt inhibitor pretreatment did not reduce the expression of Bax in melatonin pretreatment groups (Figure 5(d)). These findings indicate that melatonin may promote Akt signaling and suppress JNK signaling. Specifically, melatonin may attenuate the expression of Bax, known as an apoptotic protein through Akt activation in brain endothelial cells following OGD/R stress.

## 4. Discussion

Ischemic stroke causes oxidative stress in the brain as well as various neuropathological impairments [60]. BBB disruption is commonly observed in stroke patients [61, 62]. BBB damage is aggravated by reperfusion after ischemia [63]. ROS are generated during cerebral ischemia-reperfusion injury and lead to severe brain damage by promoting the cell apoptosis pathway [64, 65]. Also, ROS cause BBB hyperpermeability, brain edema, hemorrhage, and inflammation [66]. In the present study, we induced OGD/R injury, which is known as an appropriate* in vitro* model of stroke [67, 68], in brain endothelial cells to investigate the effect of ischemia-reperfusion injury. Recent research suggests that antioxidants attenuate oxidative damage induced by ischemia-reperfusion injury by decreasing mechanisms of ROS production [69]. Previous researches have suggested that antioxidants preserve BBB disruption and attenuate ROS generation after cerebral ischemia reperfusion* in vivo* [70–72] and* in vitro *[73, 74]. Melatonin is known as an antioxidant [75], a powerful free radical scavenger [76–78], and the cellular protector against various oxidative stress-associated diseases [79, 80]. Several studies in animals have suggested that melatonin reduces cellular damage by decreasing ROS in ischemia-reperfusion injury [46, 81, 82] and ischemia-hypoxia injury [83]. In the present study, we confirmed that melatonin reduces OGD/R-induced ROS generation in brain endothelial cells and prevents cell death of brain endothelial cells following OGD/R injury. Hypoxia causes degradation of tight junction proteins, such as Claudin 3, ZO-1 and ZO-2, and occludin [38, 39]. Several studies have demonstrated that claudins are major proteins in tight junctions [84–87], which are essential structural components of the BBB [34]. And, Claudin 5 is an important molecule that promotes disruption of the BBB in hypoxic conditions [88]. Tao et al. have demonstrated that melatonin prevents degradation of ZO-1, a tight junction protein that protects against ischemic injury in endothelial cells [89]. To determine the protective effect of melatonin on impaired BBB function caused by ischemia reperfusion, we examined Claudin 5 protein expression in brain endothelial cells following OGD/R injury. Our findings suggest that melatonin may prevent BBB disruption during ischemia-reperfusion injury by inhibiting degradation of the Claudin 5 tight junction protein. Hypoxia results in increased paracellular permeability [38, 90–92], leading to formation of cerebral edema [93]. Hypoxia induces the expression of VEGF [94–97], which is considered as one of the most important factors that stimulates the formation of new blood vessels [94, 95]. VEGF increases the permeability of blood vessels [92, 98, 99] and leads to vasogenic edema [100–103]. Several studies have demonstrated that VEGF increases BBB permeability [99], while inhibition of VEGF reduces BBB permeability [43]. Melatonin protects BBB hyperpermeability and reduces brain edema in ischemic stroke [104, 105]. Also, recent research has shown that melatonin reduces expression of VEGF in hypoxic damage [53, 106–108]. In the present study, our results showed that melatonin reduced the expression of VEGF in brain endothelial cells following OGD/R-induced injury. In oxidative stress, ROS acts as an important mediator to activate the MAPK pathway [23, 24]. The phosphatidylinositol-3-kinase/protein kinase B (PI3K/Akt) signaling pathway is considered to be one of the cell survival pathways [109]. Many researches have demonstrated that Akt plays a major role in protection from cell death under oxidative stress [110–115] and attenuates ROS production, which protects cells [116]. In brain endothelial cells, Akt enhances cell survival and inhibits apoptosis [117–119]. Melatonin promotes Akt signaling to protect cells in response to stress [120]. In the present study, our result showed that melatonin enhanced Akt activation following OGD/R injury. This finding may indicate that melatonin protects brain endothelial cells via Akt activation in the setting of ischemia-reperfusion injury. In addition, Akt can protect cellular apoptosis by regulating a proapoptotic protein such as Bax [121–124]. Several studies demonstrated that melatonin may regulate the Bax expression and may be involved in the apoptosis signaling [125, 126]. In the present study, our results showed that melatonin may regulate the Bax expression through regulating Akt activation. Considering that Bax is the proapoptotic protein, melatonin may protect the apoptosis of brain endothelial cells through suppressing the expression of Bax in response to hypoxia and reperfusion stress. JNK signaling contributes to cellular apoptosis triggered by various stresses, including oxidized LDL, proinflammatory cytokines, or high glucose [127–129]. Specifically, excessive ROS generation is closely linked to JNK activation [130–132]. JNK activation triggers the mitochondrial apoptotic pathway [133, 134] and disrupts the BBB [135]. Several studies have shown that JNK inhibitors exert protective effects against ischemic injury in a rodent model [136–139]. In the present study, our findings suggest that melatonin attenuates JNK activation in OGD/R-exposed brain endothelial cells. This result indicates that melatonin may inhibit the death of brain endothelial cells via JNK suppression. In conclusion, melatonin protects brain endothelial cells against ischemic-reperfusion injury by reducing the production of ROS, by preserving tight junction proteins, by attenuating expression of VEGF, and by regulating Akt activation and JNK suppression. Hence, this study suggests that melatonin may play as the protector on brain endothelial cells under brain hypoxic injury such as stroke. For application to the patients with stroke, this study has many limitations because of confirmation only* in vitro* study. However, these findings may provide the basic data for the further study on stroke.
